# Prevalence and predictors of psychological distress among Chinese male adults: A cross-sectional study in a large general population – CORRIGENDUM

**DOI:** 10.1017/gmh.2026.10219

**Published:** 2026-05-29

**Authors:** Tianya Hou, Xiaofei Mao, Chunyan Ni, Jianguo Zhang, Wenxi Deng, Wei Li, Ruike Zhang

**Affiliations:** 1 Faculty of Psychology, Naval Medical University, China; 2 Suzhou Hospital, Affiliated Hospital of Medical School, Nanjing University, China; 3 Nanjing University Medical School Affiliated Nanjing Drum Tower Hospital Department, China

The authors regret that some author information was incorrect in the published article.

The published author order was as follows: Tianya Hou, Chunyan Ni, Xiaofei Mao, Jianguo Zhang, Wenxi Deng, Wei Li and Ruike Zhang

The correct author order is: Tianya Hou, Xiaofei Mao, Chunyan Ni, Jianguo Zhang, Wenxi Deng, Wei Li and Ruike Zhang

The affiliation for Ruike Zhang and Wenxi Deng as published: Naval Medical University, China.

The correct affiliation for Ruike Zhang and Wenxi Deng is: Faculty of Psychology, Naval Medical University, China.

The contribution statement as published read: T.H., C.N., and X.M. these authors contributed equally to the work.

The correct contribution statement should read: T.H., X.M., and C.N. these authors contributed equally to the work.

The corresponding author information as published: Ruike Zhang; Email: zrk_2015@163.com

The correct corresponding author information is: Wei Li; Email: 18994318368@189.cn and Ruike Zhang; Email: zrk_2015@163.com

The article has been updated.
