# Prevalence and predictors of psychological distress among Chinese male adults: A cross-sectional study in a large general population – CORRIGENDUM

**DOI:** 10.1017/gmh.2026.10264

**Published:** 2026-07-14

**Authors:** Tianya Hou, Xiaofei Mao, Chunyan Ni, Jianguo Zhang, Wenxi Deng, Wei Li, Ruike Zhang

**Affiliations:** 1 Faculty of Psychology, Naval Medical University, China; 2 Nursing Department, Suzhou Hospital, Affiliated Hospital of Medical School of Nanjing University, Suzhou, China; 3 Health Management Center, Suzhou Hospital, Affiliated Hospital of Medical School of Nanjing University, Suzhou, China

The authors regret that some author information was incorrect in the published article.

Chunyan Ni affiliation as published: Suzhou Hospital, Affiliated Hospital of Medical School, Nanjing University, China.

Chunyan Ni correct affiliation: Nursing Department, Suzhou Hospital, Affiliated Hospital of Medical School of Nanjing University, Suzhou, China.

Wei Li affiliation as published: Nanjing University Medical School Affiliated Nanjing Drum Tower Hospital Department, China.

Wei Li correct affiliation: Health Management Center, Suzhou Hospital, Affiliated Hospital of Medical School of Nanjing University, Suzhou, China.

The article has been corrected.
