# Supplementary material for: Prevalence and predictors of psychological distress among Chinese male adults: A cross-sectional study in a large general population
Source: Glob Ment Health (Camb). 2026 Feb 19;13:e46. doi: 10.1017/gmh.2025.10114 (PMC13150779; doi:10.1017/gmh.2025.10114)
Supplement: Hou et al. supplementary material [file S2054425125101143sup001.docx]

**Table S1 Odds ratios (95%CI) for associations of perceived social support with depression, anxiety and stress stratified by demographic variables**

| **Perceived social support** | | **Depression** | **Anxiety** | **Stress** |
| --- | --- | --- | --- | --- |
|  |  | **COR and 95% CI** | **COR and 95% CI** | **COR and 95% CI** |
| Age: young group |  |  |  |  |
|  | Low-to-fair social support | 7.265(6.012-8.779) | 4.328(3.715-5.043) | 4.036(3.325-4.898) |
|  | High social support | 1 | 1 | 1 |
| Age: middle-aged group |  |  |  |  |
|  | Low-to-fair social support | 6.142(4.701-8.026) | 4.238(3.314-5.419) | 3.313(2.526-4.346) |
|  | High social support | 1 | 1 | 1 |
| Marital Status: Unmarried |  |  |  |  |
|  | Low-to-fair social support | 7.662(6.205-9.462) | 4.69(3.958-5.558) | 4.324(3.478-5.374) |
|  | High social support | 1 | 1 | 1 |
| Marital Status: Married |  |  |  |  |
|  | Low-to-fair social support | 5.977(4.77-7.491) | 3.819(3.124-4.67) | 3.259(2.592-4.097) |
|  | High social support | 1 | 1 | 1 |
| Only Child: Yes |  |  |  |  |
|  | Low-to-fair social support | 8.041(6.206-10.418) | 5.373(4.301-6.713) | 4.436(3.417-5.76) |
|  | High social support | 1 | 1 | 1 |
| Only Child: No |  |  |  |  |
|  | Low-to-fair social support | 6.088(5.037-7.358) | 3.788(3.23-4.443) | 3.381(2.781-4.112) |
|  | High social support | 1 | 1 | 1 |
| Children Situation: No child |  |  |  |  |
|  | Low-to-fair social support | 7.033(5.799-8.53) | 4.341(3.71-5.078) | 4.001(3.283-4.877) |
|  | High social support | 1 | 1 | 1 |
| Children Situation: One child or more |  |  |  |  |
|  | Low-to-fair social support | 6.567(5.083-8.483) | 4.276(3.392-5.39) | 3.433(2.648-4.452) |
|  | High social support | 1 | 1 | 1 |
| Educational Level:High schoold or below |  |  |  |  |
|  | Low-to-fair social support | 7.033(5.799-8.53) | 4.341(3.71-5.078) | 4.001(3.283-4.877) |
|  | High social support | 1 | 1 | 1 |
| Educational Level:College or above |  |  |  |  |
|  | Low-to-fair social support | 6.567(5.083-8.483) | 4.276(3.392-5.39) | 3.433(2.648-4.452) |
|  | High social support | 1 | 1 | 1 |
| Place of residence：Rural |  |  |  |  |
|  | Low-to-fair social support | 6.415(5.334-7.715) | 4.025(3.446-4.703) | 3.534(2.927-4.266) |
|  | High social support | 1 | 1 | 1 |
| Place of residence：Urban |  |  |  |  |
|  | Low-to-fair social support | 7.118(5.405-9.373) | 4.707(3.719-5.959) | 4.094(3.079-5.442) |
|  | High social support | 1 | 1 | 1 |
